# Knowledge and attitudes toward medical cannabis among medicine, nursing, and physiotherapy students at a Colombian university: a cross-sectional study with multivariate analysis

**DOI:** 10.1186/s42238-026-00423-x

**Published:** 2026-03-23

**Authors:** Juan Fernando Bedoya Sandoval, Luz Dary Arroyo Valencia, Pedro Andrés Molano Agudelo

**Affiliations:** Santiago de Cali University, Palmira Campus, Colombia

**Keywords:** Medical cannabis, Knowledge, Attitudes, Health students, Multivariate analysis, Cross-sectional studies, Universities

## Abstract

**Background:**

Medical cannabis has gained increasing recognition for its therapeutic potential in various clinical conditions. However, knowledge and attitudes toward its medical use among future health professionals in Colombia remain limited and insufficiently investigated.

**Objective:**

To assess the levels of knowledge and attitudes regarding medical cannabis among Medicine, Nursing, and Physiotherapy students at a private Colombian university, identify sociodemographic predictors of higher knowledge and favorable attitudes. The findings are then discussed in the context of national and international public health and regulatory frameworks, highlighting potential areas for educational consideration.

**Methods:**

A cross-sectional study was conducted among 658 undergraduate students selected through simple random sampling stratified by academic program and semester, using official enrollment lists as the sampling frame. A validated questionnaire assessed sociodemographics, knowledge, and attitudes. Descriptive statistics, chi-square tests, and binary logistic regression identified predictors of higher knowledge and favorable attitudes (*p* < 0.05; 95% CI). Public health implications were analyzed in relation to WHO, PAHO, and UNODC recommendations, as well as Colombian health and education policy.

**Results:**

Overall, 63% reported moderate to substantial knowledge, though significant gaps persisted: 69% demonstrated low self-perceived knowledge regarding therapeutic dosage, 92% lacked awareness of legal regulations, and 60% showed insufficient knowledge about pharmaceutical forms. Attitudes toward the medical use of cannabis were predominantly favorable: 75% supported expanded legalization and 89% endorsed academic training. Multivariate analysis showed that being a medical student (OR: 2.8; 95% CI: 1.9–4.3) and being in advanced semesters (OR: 1.6; 95% CI: 1.1–2.5) predicted higher knowledge. Favorable attitudes were predicted by higher knowledge (OR: 3.4; 95% CI: 2.3–5.0) and clinical rotation experience (OR: 1.9; 95% CI: 1.3–2.8).

**Conclusions:**

Results suggest that incorporating medical cannabis education into health curricula may be beneficial, although additional studies are needed to confirm this recommendation in broader contexts.

**Supplementary Information:**

The online version contains supplementary material available at 10.1186/s42238-026-00423-x.

## Introduction

Medical cannabis refers to products derived from *Cannabis sativa*, containing active components such as Δ9-tetrahydrocannabinol (THC) and cannabidiol (CBD), which interact with CB1 and CB2 receptors in the central and peripheral nervous system and immune cells (Ledezma-Morales et al. 2020). Evidence from controlled clinical trials and systematic reviews has supported the therapeutic use of medical cannabis for selected clinical conditions including epilepsy, multiple sclerosis, chemotherapy-induced nausea and vomiting, and chronic pain (Bridgeman and Abazia 2017; Mayo Clinic, 2024).

In Colombia, regulatory frameworks such as Law 1787 of 2016 and Decree 811 of 2021 established legal mechanisms for medical cannabis production, distribution, and prescription under controlled conditions (Congreso de Colombia, 2016). Nevertheless, despite legalization, the integration of medical cannabis education in undergraduate health curricula remains limited.

International studies have highlighted notable gaps in knowledge and attitudes among health students regarding medical cannabis, particularly in areas such as dosage, legal frameworks, potential risks, and appropriate indications (Zajicek et al. 2003; Devinsky et al. 2017; Sokratous et al. 2021). In Latin America, Lopera-Londoño et al. (Lopera-Londoño et al. 2018) identified insufficient knowledge and cautious attitudes among pharmacy and medical students in Colombia, suggesting the need for curricular strengthening. In Colombia, recent reports from the Ministry of Health indicate an increase in the number of medical cannabis prescriptions issued in 2024, reflecting the growing clinical relevance of this therapeutic approach. However, previous studies among Latin American healthcare professionals have consistently identified substantial knowledge gaps regarding dosage, clinical indications, and legal aspects (Velasco Ramírez and Godínez Tamayo 2020; Gómez-Echeandía et al. 2022).

According to the World Health Organization (WHO) (World Health Organization 2024), Pan American Health Organization (PAHO) (Pan American Health Organization 2023), and the United Nations Office on Drugs and Crime (UNODC) (United Nations Office on Drugs and Crime 2024), public health frameworks for medical cannabis regulation should be accompanied by robust educational strategies for health professionals to ensure evidence-based, safe therapeutic use. In Colombia, the Ministry of National Education (MinEducación) and the Ministry of Health (Minsalud) have promoted guidelines and processes to improve health training; however, official evidence shows that these actions have been mostly guided and participatory, with guidelines and conceptual frameworks under development rather than detailed national curriculum guides for all undergraduate programs (Ministry of National Education 2015).

Given this context, this study aimed to evaluate the levels of knowledge and attitudes regarding medical cannabis among Medicine, Nursing, and Physiotherapy students at a private university in Palmira, Colombia. Additionally, the study sought to identify sociodemographic predictors of higher knowledge and favorable attitudes using multivariate analysis and to discuss implications aligned with international public health recommendations and Colombian education policy.

## Methods

### Study design and period

A cross-sectional study was conducted at Universidad Santiago de Cali, Palmira campus, between March and September 2024. The reporting of this study follows the CHERRIES guidelines to ensure comprehensive and transparent reporting of the web-based survey methods (see Supplementary Material, Appendix 1).

### Population and sample

The target population consisted of 1,395 undergraduate students officially enrolled from first to tenth semester in the Medicine (*n =* 681), Nursing (*n =* 404), and Physiotherapy (*n =* 310) programs during the academic period 2025–1.

### Sample size calculation

The required sample size was estimated a priori based on the expected prevalence of high knowledge about medical cannabis. Since no previous Colombian study had specifically assessed knowledge levels in this population using the same instrument, we considered the most conservative scenario. Assuming an expected proportion of 50% (*p =* 0.5) of students with high knowledge, with a 5% margin of error and a 95% confidence level (Z = 1.96), the minimum required sample size was 384 participants. To account for potential missing data and incomplete responses, the sample was oversampled by 20%, resulting in a target of 461 participants. The final achieved sample was 658 participants, exceeding the minimum required size and providing adequate statistical power for multivariate analysis.

### Sampling procedure

A stratified simple random sampling strategy was applied, using the official enrollment lists obtained from the Academic Registry as the sampling frame. Stratification was performed by academic program (Medicine, Nursing, Physiotherapy) and semester (1st to 10th) to ensure proportional representation. Within each stratum, participants were randomly selected using a random number generator. The overall response rate was 47.2% (658/1,395). Program-specific response rates were 56% in Medicine (381/681), 39% in Nursing (158/404), and 38% in Physiotherapy (118/310).

### Inclusion and exclusion criteria

Inclusion criteria were: (1) being officially enrolled in Medicine, Nursing, or Physiotherapy; (2) being 18 years of age or older; (3) being registered in semesters 1–10; and (4) voluntarily providing informed consent. Exclusion criteria included: (1) incomplete questionnaires (< 80% answered); (2) duplicate submissions; (3) inconsistencies identified during data validation; and (4) participation in academic mobility programs at the time of data collection.

### Variables and instruments

Data were collected using a structured, self-administered questionnaire adapted from previously validated instruments (De Santiago Moraga 2022; García Acosta 2018). The questionnaire consisted of three sections:

#### Sociodemographic variables

Six items assessed: age (continuous, in years), sex (male/female), academic program (Medicine/Nursing/Physiotherapy), semester (categorized as ≤ 4th vs. ≥ 5th), clinical rotation experience (yes/no), and religion (Catholic, Christian/Evangelical, Atheist/Agnostic, Other). Religion was collected for descriptive purposes due to its potential cultural influence on attitudes toward cannabis, but it was not included in the multivariate analysis as it was not a primary objective and preliminary bivariate analyses showed no significant association with knowledge or attitudes (*p* > 0.05) (Table 1).

**Table 1:** Sociodemographic characteristics of the population

Variable	Frequency (n)	Percentage (%)
Sex		
Female	443	67.3
Male	215	32.7
Academic program		
Medicine	381	58.0
Nursing	158	24.0
Physiotherapy	118	18.0
Age* (mean ± SD)	—	21.4 ± 2.7
Religion		
Catholic	212	45.9
Christian/Evangelical	93	20.1
Atheist/Agnostic	75	16.2
Other	82	17.8
No response	196	29.8

^*^Age is presented as mean ± standard deviation. Percentages are calculated based on the total sample (*N* = 658). "No response" includes participants who did not answer the religion item

#### Knowledge about medical cannabis

Knowledge was defined as the participant's self-reported understanding of factual information regarding the medical use of cannabis, including its legal status, therapeutic indications, safety profile, and regulatory framework. It is important to note that this measure reflects perceived knowledge rather than objectively tested knowledge, which may be subject to self-assessment bias. This construct was measured using eight items with a 4-point Likert scale ranging from 1 ("no knowledge") to 4 ("high knowledge"). Total knowledge scores were calculated by summing all items (possible range: 8 to 32), with higher scores indicating greater perceived knowledge. For bivariate and multivariate analyses, scores were dichotomized using a cutoff of ≥ 75% of the maximum score (≥ 24 points) to define "high knowledge," based on commonly used thresholds in health education research (García Acosta 2018; World Health Organization 2024). Sensitivity analyses using alternative cutoffs (70% and 80%) yielded consistent results. For the purpose of item-level analysis presented in Table 2, responses on the 4-point Likert scale were dichotomized: scores of 3 or 4 ("high knowledge") were categorized as high self-reported knowledge, while scores of 1 or 2 ("no knowledge" or "low knowledge") were categorized as low self-reported knowledge. This threshold aligns with the definition of high knowledge used for the total score.

**Table 2:** Knowledge and attitudes toward medical cannabis among health sciences students (n = 658)

Item	Response, n (%)			
Knowledge items	High knowledge		Low knowledge	
Medical cannabis is legal in Colombia	566(86.1)		92 (13.9)	
Medical cannabis can be used for the management of chronic pain	530 (80.5)		128 (19.5)	
Medical cannabis has no adverse effects	145 (22.1)		513 (77.9)	
Medical cannabis is regulated by the Ministry of Health	485 (73.8)		173 (26.2)	
Medical cannabis has abuse and dependence potential	362 (55.0)		296 (45.0)	
Medical cannabis therapeutic dosage is well established	210 (31.9)		448 (68.1)	
Pharmaceutical formulations of medical cannabis include oils, capsules, and topical preparations	263 (40.0)		395 (60.0)	
Specific regulatory risks are associated with medical cannabis prescription	53 (8.0)		605 (92.0)	

Attitude items	Disagree	Neutral		Agree
Physicians should be allowed to prescribe medical cannabis	40 (6.1)	244 (37.0)		374 (56.9)
Medical cannabis is safe when used responsibly	46 (7.0)	151 (23.0)		461 (70.0)
Medical cannabis could be harmful despite therapeutic use	165 (25.0)	447 (68.0)		46 (7.0)
Medical cannabis has been adequately researched	66 (10.0)	191 (29.0)		401 (61.0)
Medical cannabis is safe under medical prescription	26 (4.0)	165 (25.0)		467 (71.0)
Medical cannabis is safe without prescription	165 (25.0)	250 (38.0)		243 (37.0)
Medical cannabis is overused	118 (18.0)	211 (32.0)		329 (50.0)
Legalization increases non-medical use	33 (5.0)	191 (29.0)		434 (66.0)
I would support medical legalization	20 (3.0)	217 (33.0)		421 (64.0)
Medical cannabis should treat more diseases	20 (3.0)	151 (23.0)		487 (74.0)
More education on medical cannabis is needed	13 (2.0)	125 (19.0)		520 (79.0)
Health professionals should receive cannabis education	0 (0.0)	66 (10.0)		592 (90.0)

For knowledge items, "High knowledge" corresponds to a self-reported score of 3 or 4 on a 4-point Likert scale (where 1 = "no knowledge", 4 = "high knowledge"), and "Low knowledge" corresponds to scores of 1 or 2. For attitude items, "Disagree" includes strongly disagree and slightly disagree; "Agree" includes slightly agree and totally agree. Percentages may not total 100 due to rounding

#### Attitudes toward medical cannabis

Attitudes were defined as the participants' evaluative perceptions, beliefs, and predispositions toward the medical use of cannabis in clinical practice and health professional education. This construct was measured using 12 items with a 5-point Likert scale ranging from 1 ("strongly disagree") to 5 ("strongly agree"), including both positively and negatively worded statements. Negatively worded items were reverse-scored for analysis. Total attitude scores were calculated by summing all items (possible range: 12 to 60), with higher scores indicating more favorable attitudes toward medical cannabis. For bivariate and multivariate analyses, scores were dichotomized using a cutoff of ≥ 75% of the maximum score (≥ 45 points) to define "favorable attitudes."

#### Instrument adaptation process

The questionnaire was adapted from previously validated instruments (De Santiago Moraga 2022; García Acosta 2018) through a multistep process. First, the original Spanish versions were reviewed for linguistic and contextual appropriateness by a panel of three experts in public health and nursing education. Second, a pilot test was conducted with 30 students from the target population to assess clarity, comprehension, and completion time. Minor adjustments were made based on pilot feedback. Finally, internal consistency was assessed, yielding Cronbach's α values of 0.81 for the knowledge scale and 0.84 for the attitude scale.

### Data collection

The questionnaire was implemented as an online survey using the institutional digital platform Microsoft Forms. Potential participants received an invitation email containing a brief explanation of the study objectives, assurance of anonymity and confidentiality, and a personalized link to access the survey. The survey was accessible from March 1 st to September 30th. Weekly reminder emails were sent to non-respondents during the data collection period to maximize response rates. Participation was voluntary, and no incentives were offered. Informed consent was obtained electronically before participants could access the questionnaire; only those who clicked "I agree to participate" were able to proceed.

### Data analysis

Statistical analysis was performed using SPSS version 25 (IBM Corp., Armonk, NY, USA).

#### Descriptive analysis

Frequencies and percentages were calculated for categorical variables, while means and standard deviations (or medians and interquartile ranges for non-normally distributed variables) were computed for continuous variables.

#### Normality testing

The Shapiro–Wilk test was used to assess the normality of continuous variables (age, knowledge scores, attitude scores). Given the sample size, visual inspection of histograms and Q-Q plots was also performed.

#### Bivariate analysis

Associations between categorical predictors and the outcomes (high knowledge and favorable attitudes) were tested using the chi-square test or Fisher's exact test when expected cell frequencies were < 5. For continuous variables, independent samples t-tests were applied when normality assumptions were met; otherwise, the Mann–Whitney U test was used. To reduce Type I error inflation due to multiple comparisons, a Bonferroni correction was applied when appropriate.

#### Multivariate analysis

Binary logistic regression models were developed to identify independent predictors of: (1) high knowledge, and (2) favorable attitudes. Variables with *p* < 0.20 in the bivariate analysis were included as candidates for the multivariate models. The models were fitted using the Enter method to ensure theoretical and epidemiological consistency. Multicollinearity was assessed using Variance Inflation Factors (VIF), with values < 5.0 considered acceptable. Interactions between academic program and semester group were tested; non-significant interactions were removed from the final models. Model fit was evaluated using the Hosmer–Lemeshow goodness-of-fit test (acceptable if *p* > 0.05), and standardized residuals were examined. Adjusted odds ratios (AOR) with 95% confidence intervals (CI) were reported. A *p*-value < 0.05 was considered statistically significant.

#### Post-hoc power analysis

Post-hoc power estimation indicated that both logistic regression models achieved adequate power (> 0.80) to detect meaningful associations.

### Ethical considerations

This study was approved by the Ethics Committee of Universidad Santiago de Cali (Act No. 2023–18). All procedures were conducted in accordance with the principles of the Declaration of Helsinki and Colombian regulations for health research (Resolution 8430 of 1993). Participants were informed about the study objectives, the voluntary nature of participation, and their right to withdraw at any time without consequences. Anonymity and confidentiality were guaranteed; no personal identifiers were collected, and all data were stored on password-protected servers accessible only to the research team. Informed consent was obtained electronically prior to survey completion.

## Results

### Sociodemographic characteristics

A total of 658 students participated in the study, with a mean age of 21.4 ± 2.7 years. The sample was predominantly female (67.3%). Regarding academic programs, 58.0% were enrolled in Medicine, 24.0% in Nursing, and 18.0% in Physiotherapy. Most participants were in advanced academic semesters (≥ fifth semester), and 65% reported having completed clinical rotations.

### Knowledge about medical cannabis

Overall, 63% of students demonstrated moderate to high levels of knowledge regarding medical cannabis. Item-level analysis revealed adequate awareness of its legal status in Colombia (86.1%) and its regulation by the Ministry of Health (73.8%). Additionally, most participants reported high self-perceived knowledge regarding its therapeutic role in chronic pain management (80.5%).

However, substantial knowledge gaps were identified in clinically relevant domains. Only 55.0% reported high self-perceived knowledge regarding its potential for abuse and dependence, and just 22.1% reported high knowledge about possible adverse effects, indicating limited understanding of safety considerations. Furthermore, complementary analyses showed low self-reported knowledge related to therapeutic dosage (68.1% low knowledge), pharmaceutical formulations (60.0% low knowledge), and specific regulatory risks (92.0% low knowledge), highlighting critical deficits in practical clinical competency.

Higher knowledge levels were significantly more frequent among medical students (72%) and those enrolled in advanced academic semesters (68%), compared with students from other programs and earlier semesters (*p* < 0.001). Specifically, high knowledge was observed in 72% (274/381) of medical students, 48% (76/158) of nursing students, and 41% (48/118) of physiotherapy students.

### Attitudes toward medical cannabis

Attitudes toward medical cannabis were predominantly favorable. Approximately 75% of students supported the expansion of medical cannabis legalization for additional therapeutic indications, and 70% agreed that its use is safe when prescribed by qualified healthcare professionals. Notably, 89% of respondents expressed strong support for the inclusion of formal medical cannabis education within undergraduate health sciences curricula. Favorable attitudes were significantly more prevalent among students with prior clinical rotation experience (82%) and those with higher levels of knowledge (85%) (*p* < 0.001).

### Missing data

Missing data were minimal for most variables (< 2% for any single item), with the exception of religion, which had 196 non-responses (29.8%). This variable was collected for descriptive purposes only and was not included in the main analyses. Questionnaires with less than 80% completion (*n =* 12, 1.8%) were excluded from the analysis. For the remaining sample, missing values were handled using pairwise deletion in descriptive analyses and listwise deletion in regression models, as the low missingness rate for key variables (< 5%) is unlikely to introduce bias (Little and Rubin 2019).

### Multivariate analysis

In the multivariate logistic regression model, being enrolled in the Medicine program was independently associated with a higher likelihood of having high knowledge about medical cannabis (AOR = 2.80; 95% CI: 1.90–4.30; *p* < 0.001). Similarly, students in advanced academic semesters (≥ fifth semester) showed increased odds of high knowledge compared to those in earlier semesters (AOR = 1.60; 95% CI: 1.10–2.50; *p =* 0.022). Age and sex were not independently associated with high knowledge after adjustment.

Regarding favorable attitudes toward the medical use of cannabis, higher knowledge levels emerged as the strongest independent predictor (AOR = 3.40; 95% CI: 2.30–5.00; *p* < 0.001). In addition, having completed clinical rotations was significantly associated with favorable attitudes (AOR = 1.90; 95% CI: 1.30–2.80; *p =* 0.001).

Full model estimates are presented in Table 3

**Table 3:** Predictors of high knowledge and favorable attitudes toward medical cannabis (binary logistic regression analysis)

Outcome	Variable	OR (95% CI)	*p*-value	AOR (95% CI)	*p*-value
High Knowledge	Medical program (Medicine vs others)	3.12 (2.20–4.43)	< 0.001	2.80 (1.90–4.30)	< 0.001
	Advanced semester (≥ 5th vs ≤ 4th)	1.84 (1.31–2.60)	< 0.001	1.60 (1.10–2.50)	0.022
	Age (> 22 vs ≤ 22 years)	1.42 (1.01–2.01)	0.046	1.21 (0.84–1.74)	0.302
	Sex (male vs female)	1.18 (0.86–1.63)	0.295	1.09 (0.78–1.54)	0.612
Favorable Attitudes	High knowledge (yes vs no)	3.92 (2.76–5.56)	< 0.001	3.40 (2.30–5.00)	< 0.001
	Clinical rotations (yes vs no)	2.14 (1.55–2.96)	< 0.001	1.90 (1.30–2.80)	0.001

Model fit: For the high knowledge model: Cox & Snell R^2^ = 0.18, Nagelkerke R^2^ = 0.25. For the favorable attitudes model: Cox & Snell R^2^ = 0.22, Nagelkerke R^2^ = 0.31

Multivariate models adjusted for age, sex, academic program, semester, and clinical rotation experience

Variables with *p* < 0.20 in bivariate analysis were included in the adjusted models

Hosmer–Lemeshow goodness-of-fit test indicated adequate model fit (*p* > 0.05)

*OR* crude odds ratio, *AOR* adjusted odds ratio

## Discussion

This study revealed that while health sciences students at a Colombian university hold predominantly favorable attitudes toward medical cannabis, significant knowledge gaps persist in clinically relevant areas such as therapeutic dosage, legal regulations, and pharmaceutical formulations. These findings suggest a disconnect between students' willingness to embrace medical cannabis and their actual preparedness to use it safely in future clinical practice.

The level of knowledge observed (63% with moderate to high knowledge) is comparable to that reported in similar studies from other contexts. For instance, Sokratous et al. (Sokratous et al. 2021) found that Greek nursing students demonstrated moderate knowledge but with similar gaps in safety and regulatory aspects, while Bawa et al. (Bawa et al. 2022) reported that Australian general practitioners lacked confidence in dosing and prescribing. In Latin America, Lopera-Londoño et al. (Lopera-Londoño et al. 2018) also identified insufficient knowledge among Colombian pharmacy and medical students, particularly regarding legal frameworks. The consistency of these findings across diverse settings underscores a global challenge: the integration of medical cannabis education into health curricula has not kept pace with legislative changes and clinical demand (Zolotov et al. 2021).

The multivariate analysis showed that being enrolled in the medicine program and being in advanced academic semesters were independently associated with significantly higher odds of high knowledge regarding medical cannabis (AOR = 2.80 and 1.60, respectively). These associations are consistent with previous studies (De Santiago Moraga 2022; Zolotov et al. 2021) and likely reflect greater exposure to pharmacology and clinical training, reinforcing the role of structured curricula in building competency.

From a public health and educational policy perspective, these findings highlight the need to incorporate medical cannabis training into health professions curricula. International organizations such as the WHO (World Health Organization 2024), PAHO (Pan American Health Organization 2023), and UNODC (United Nations Office on Drugs and Crime 2024) have emphasized the importance of evidence-based education to ensure safe therapeutic use. In Colombia, although Law 1787 of 2016 and Decree 811 of 2021 permit medical cannabis prescription (Congreso de Colombia, 2016), undergraduate programs lack standardized content on this topic. Recent evidence from Restrepo et al. (Restrepo et al. 2023) demonstrates that targeted educational interventions can significantly improve students' knowledge and attitudes, suggesting that similar initiatives could be effective across health disciplines. Moreover, experiences from Brazil (Rodrigues et al. 2024; Viana et al. 2024; Barreto 2025) underscore the value of both formal and informal educational strategies, including patient-led advocacy and digital platforms, in disseminating knowledge about medicinal cannabis. However, given the single-center design, these implications should be considered preliminary and require confirmation through multicenter studies.

This study has several strengths, including its large sample size, stratified random sampling, and use of a validated instrument. However, the findings should be interpreted considering the limitations outlined in the corresponding section. Future multicenter, longitudinal studies are needed to confirm these findings, evaluate the impact of curricular interventions, and explore discipline-specific educational needs across health professions.

In conclusion, this study provides baseline evidence that Colombian health sciences students hold favorable attitudes toward medical cannabis but lack essential knowledge for its safe use. These findings support the call for integrating formal medical cannabis education into undergraduate health curricula, though further research is required to determine the most effective educational strategies and their long-term impact on patient care.

### Limitations

Although a probabilistic sampling strategy was planned, participation was ultimately voluntary, which may have introduced self-selection bias and limited representativeness of the sample.

Several limitations should be considered when interpreting the findings of this study. First, the cross-sectional design limits the ability to establish causal relationships between sociodemographic or academic variables and levels of knowledge or attitudes toward medical cannabis. Consequently, observed associations should be interpreted as correlational. Longitudinal or cohort studies are recommended to examine temporal changes and the impact of educational interventions over time, as suggested in previous research (Zolotov et al. 2021). Second, the study was conducted at a single private university, which introduces potential selection bias and limits the external validity of the findings. Students enrolled in private institutions may differ from those in public universities in terms of academic exposure, socioeconomic background, and access to curricular resources. Therefore, the results may not be fully generalizable to all health sciences students in Colombia or other Latin American settings. Multicenter studies including public and private universities, as well as postgraduate trainees from diverse geographic regions, are warranted to enhance generalizability (Restrepo et al. 2023).

Third, data were collected through self-administered questionnaires, which are inherently susceptible to social desirability and response bias, particularly when addressing topics related to substance use and regulation. Although anonymity and voluntary participation were ensured to minimize this risk, some degree of overreporting of favorable attitudes or perceived knowledge cannot be excluded. Incorporating qualitative approaches, such as focus groups or in-depth interviews, could provide deeper insights and help triangulate self-reported findings.

Fourth, despite the use of multivariate logistic regression, residual confounding by unmeasured variables remains possible. Factors such as personal or family experience with cannabis use, religious beliefs, prior informal exposure to cannabis-related information, or previous participation in formal training programs were not assessed and may have influenced both knowledge levels and attitudes. Including these variables in future analytical models could improve explanatory power and yield a more comprehensive understanding of determinants.

Fifth, the assessment of knowledge relied on self-reported perceptions rather than objective testing. This approach may overestimate or underestimate actual knowledge, as participants might inaccurately gauge their understanding of medical cannabis. Future studies should incorporate validated knowledge tests with correct/incorrect response options to obtain more accurate estimates of competency.

Despite these limitations, this study provides valuable baseline evidence on knowledge and attitudes toward medical cannabis among health sciences students in Colombia. The findings contribute to the growing body of evidence supporting the need for structured educational strategies and may inform future curricular development and public health policy initiatives.

## Conclusions

This single-center study suggests that Colombian health sciences students hold favorable attitudes toward medical cannabis but lack essential knowledge for its safe use, particularly in areas such as dosage, legal regulations, and pharmaceutical formulations. Enrollment in medicine and advanced academic semesters were associated with higher knowledge, while higher knowledge and clinical experience predicted favorable attitudes.

These findings support the potential value of integrating formal medical cannabis education into undergraduate health curricula, though multicenter and longitudinal studies are needed to confirm these observations and guide evidence-based educational strategies.

## Supplementary Information

Supplementary Material 1.

## Data Availability

The datasets used and analyzed during the current study are available from the corresponding author upon reasonable request.
