# Supplementary material for: Knowledge and attitudes toward medical cannabis among medicine, nursing, and physiotherapy students at a Colombian university: a cross-sectional study with multivariate analysis
Source: J Cannabis Res. 2026 Mar 23;8:61. doi: 10.1186/s42238-026-00423-x (PMC13130541; doi:10.1186/s42238-026-00423-x)
Supplement: Supplementary file 1 — Supplementary Material 1. [file 42238_2026_423_MOESM1_ESM.docx]

**Supplementary Material, Appendix 1, Table 1. CHERRIES Checklist.**

| **Item** | **Description** | **How it was addressed in this study** |
| --- | --- | --- |
| **Design** | Describe the survey design | Cross-sectional online survey |
| **Institutional review board** | IRB approval obtained | Yes (Act No. 2023-18) |
| **Informed consent** | How consent was obtained | Electronic consent before accessing the survey |
| **Data protection** | Anonymity and confidentiality ensured | No personal identifiers collected; data stored on password-protected servers |
| **Target population** | Describe the target population | Medicine, Nursing, and Physiotherapy students at Universidad Santiago de Cali, Palmira campus |
| **Sampling frame** | How participants were identified | Official enrollment lists from Academic Registry |
| **Sample size calculation** | Was sample size calculated? | Yes (n = 384 minimum; 461 target; 658 achieved) |
| **Survey administration** | Platform used | Microsoft Forms |
| **Survey dates** | When was the survey open? | March and September 2024 |
| **Invitations** | How were participants invited? | Email invitation with personalized link |
| **Reminders** | Were reminders sent? | Weekly reminder emails to non-respondents |
| **Response rate** | Number of participants who completed the survey / number invited | 658 / 1,395 = 47.2% |
| **Preventing multiple entries** | How were duplicates prevented? | IP addresses were checked for duplicates [o: Cookies were used to prevent multiple submissions] |
| **Completeness check** | Were incomplete questionnaires handled? | Questionnaires with <80% completion were excluded |
| **Statistical analysis** | Software used | SPSS version 25 |
